# The complete mitochondrial genome of *Hylarana latouchii* (Anura: Ranidae) and its phylogenetic analysis

**DOI:** 10.1080/23802359.2021.1942260

**Published:** 2021-06-22

**Authors:** Yi-Jia Sun, Yue-Yue Zheng, Wei-Cheng Zheng, Zhi-Hua Lin, Fen Qiao

**Affiliations:** a College of Ecology, Laboratory of Amphibian Diversity Investigation, Lishui University, Lishui, China; b Administration Bureau of Zhejiang, Jiulongshan National Nature Reserve, Suichang, China

**Keywords:** *Hylarana latouchii* (Boulenger, 1899), mitochondrial genome, phylogenetic analysis, next-generation sequencing

## Abstract

We reported the complete mitochondrial genome (mitogenome) of broad-folded frog (*Hylarana latouchii*). This mitogenome is 17,007 bp in size and consists of 13 protein-coding genes, 22 transfer RNAs, two ribosomal RNAs, and one non-coding sequence (D-loop). The total composition was 58.54% A + T and 41.46% G + C (T: 29.31%, C: 27.33%, A: 29.23%, and G: 14.13%). The phylogenetic analysis revealed that *H. latouchii* formed a clade with other two species of genus *Hylarana*. This mitogenomic sequence of *H. latouchii* provides useful data to study its population genetics and phylogeography.

The broad-folded frog, *Hylarana latouchii* (Boulenger, 1899), is an endemic species in southeastern China (Amphibia China 2021). *Hylarana* containing 12 species is distributed in tropical and subtropical Asia. The phylogenetic relationship and classification of this genus still remains controversial (Oliver et al. 2015). Here, we determined the complete mitochondrial genome (mitogenome) of *H. latouchii*, and compared the sequence with other species of Ranidae.

The specimen of *H. latouchii* (species voucher: LSU20200422001ZL) was collected in Jiulongshan National Nature Reserve (28.37°N, 118.90°E), Zhejiang, China and deposited in −80 °C at Laboratory of Amphibian Diversity Investigation, Lishui University, China (Fen Qiao, qiaofen121@163.com). We have long-term cooperation to investigate and protect amphibians in this area and obtain permission for sampling and sample storage. We used EasyPure Genomic DNA Kit (TransGen Biotech Co., Beijing, China) to extract total DNA from muscle tissue of *H. latouchii*. The mitogenome sequences of *H. latouchii* were acquired by Illumina NovaSeq 6000 platform (Novogene Bioinformatics Technology Co. Ltd., Tianjin, China) for PE 2 × 150 BP sequencing. We used NOVO Plasty 3.7 (Dierckxsens et al. 2017) to *de novo* assemble a closed-circular mitogenome of *H. latouchii* and used MITOS WebServer (Matthias et al. 2013) and tRNAscan-SE (Lowe and Chan 2016) to annotate the mitogenome.

The complete mitogenome of *H. latouchii* (GenBank accession number MT702387) is 17,007 bp in size and consists of 37 genes, containing 13 protein-coding genes (PCGs) (ATP6, ATP8, COI-III, ND1-6, ND4L, and CYTB), 22 transfer RNA (tRNA) genes, two ribosomal RNA (rRNA) genes (16S and 12S), and a control region (D-loop), which is similar to other mitogenomes of most vertebrates, such as *H. guentheri* is 18,698 bp in length and consists of 37 genes (Jiang et al. 2020). The longest one is ND5 (1803 bp), and the shortest one is ATP8 (165 bp) in the 13 PCGs which are longer than the previous report of Xiao et al. (2019). A majority of PCGs start with an ATG codon except ND2 begins with ATT, and COI starts with GTG. Whereas, in previous report, COI starts with ATT, and ND6 starts with CCT (Xiao et al. 2019). The overall base composition for the mitogenome of *H. latouchii* was as follows: 29.23% for A, 29.31% for T, 27.33% for C, and 14.13% for G, but in previous report, the content of G was 27.3% (Xiao et al. 2019).

A Bayesian tree (with 1,000,000 generations, sampling every 1000 generations and discarding 1000 trees as burn-in) containing the 13 PCGs in mitogenomes of 17 species (*Occidozyga martensii* serve as outgroup taxa) was constructed by MrBayes v3.2.2 software (Ronquist et al. 2012). The phylogenetic relationship of the 17 species is shown in Figure 1. The *H. latouchii* is sister to *H. kreffti* which is concordant with previous research (Xiao et al. 2019). However, the *H. guentheri* is close with *Nidirana adenopleura* (Jiang et al. 2020) and not clustered with other three species in this genus (Figure 1). Our data would be useful for subsequent research about mitogenome evolution and phylogenetic relationships in *Hylarana*.

**Figure 1.**
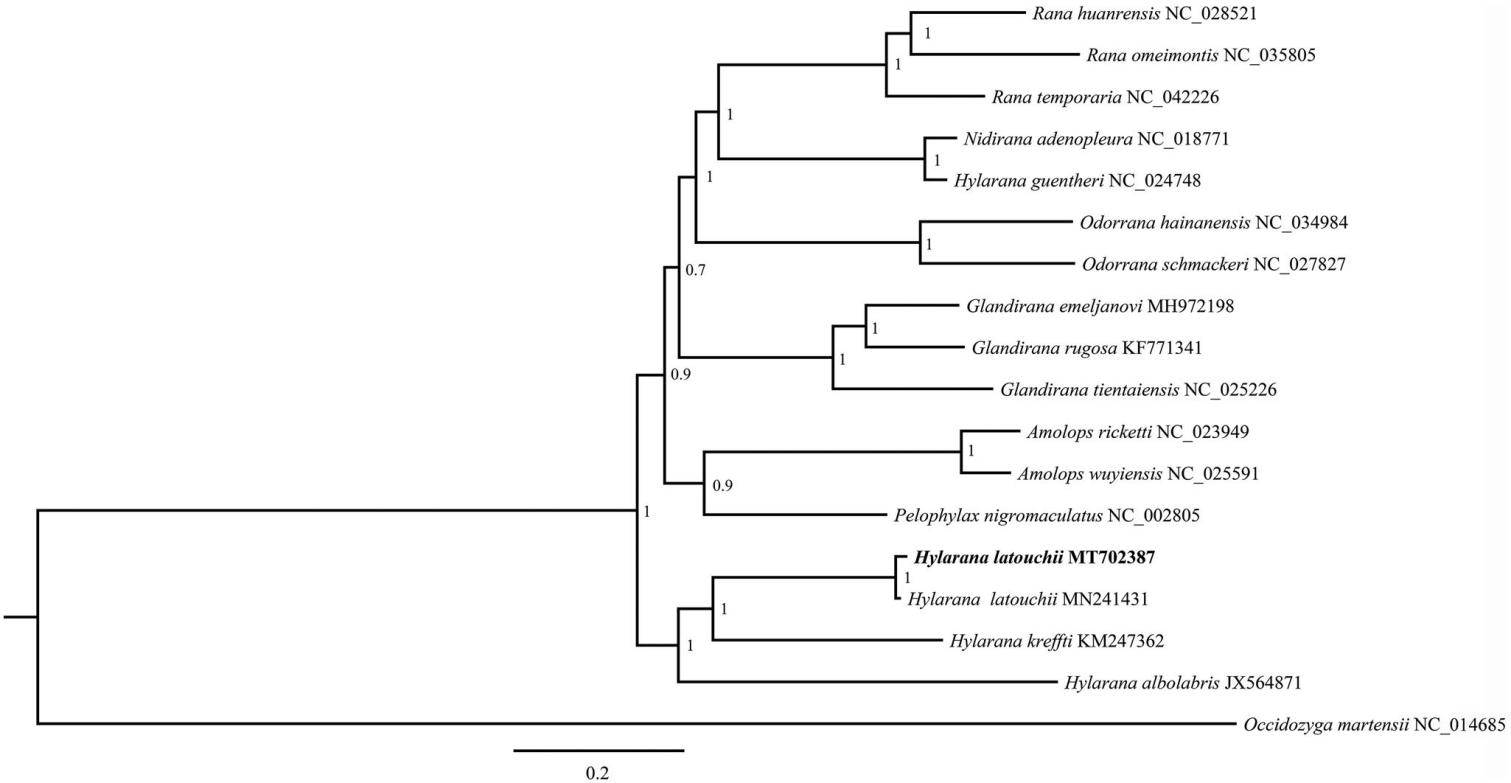
The phylogenetic tree of *Hylarana latouchii* and other 16 species was constructed by Bayesian inference (BI) method based on 13 PCGs.

## Data Availability

The mitogenome data supporting this study are openly available in GenBank at https://www.ncbi.nlm.nih.gov/nuccore/MT702387. Reference number [accession number: MT702387]. BioSample and SRA accession numbers are https://www.ncbi.nlm.nih.gov/biosample/SAMN15367072, https://www.ncbi.nlm.nih.gov/sra/SRR12088660, respectively.
